# Supplementary material for: European population trends and current conservation status of an endangered steppe-bird species: the Dupont’s lark Chersophilus duponti
Source: PeerJ. 2018 Sep 19;6:e5627. doi: 10.7717/peerj.5627 (PMC6151120; doi:10.7717/peerj.5627)
Supplement: Supplemental Information 2 — Wald-tests and associated p-values (p) are shown. For each period, the annual change rate, the associated 95% Confidence Interval (CI95%) and trend classification attending to TRIM criteria (TRIM Trend; Pannekoek and Van Strien 2006a) are shown. [file peerj-06-5627-s002.docx]

| **Period** | **Wald-test** | **p** | **Annual change rate (%)** | **CI95%** | **TRIM Trend** |
| --- | --- | --- | --- | --- | --- |
| 2004 – 2006 | 13.36 | < 0.001 | -18.3 | [-27.1; -9.4] | Steep decline |
| 2006 – 2007 | 10.74 | < 0.01 | +33.1 | [+5.3; +60.8] | Strong increase |
| 2007 – 2009 | 3.01 | < 0.1 | +4.9 | [-4.4; +14.2] | Uncertain |
| 2009 – 2010 | 35.86 | < 0.001 | -42.1 | [-50.1;-34.0] | Steep decline |
| 2010– 2012 | 44.63 | < 0.05 | +11.4 | [+3.5; +19.2] | Moderate increase |
| 2012 – 2015 | 10.57 | < 0.001 | -5.8 | [-10.2; -1.5] | Moderate decline |
