# Supplementary material for: European population trends and current conservation status of an endangered steppe-bird species: the Dupont’s lark Chersophilus duponti
Source: PeerJ. 2018 Sep 19;6:e5627. doi: 10.7717/peerj.5627 (PMC6151120; doi:10.7717/peerj.5627)
Supplement: Supplemental Information 3 — The number of males at the first time-point (Nt0) and the first year of the temporal series (t0), are shown. In addition, the year when the population was considered extinct or the second year without detecting the species (ti), is indicated. [file peerj-06-5627-s003.docx]

| **Autonomous Community** | **Province** | **Population** | **N_t0_** | **t_0_** | **t_i_** |
| --- | --- | --- | --- | --- | --- |
| Andalusia | Almeria | Filabres-Baza-Cerro Villegas | 1 | 2009 | 2012 |
| Andalusia | Almeria | Sierra de Gador-La Campita | 5 | 2004 | 2015 |
| Andalusia | Almeria | Sierra de Gador-Llano de la Mota | 1 | 2008 | 2010 |
| Andalusia | Almeria | Tabernas-Sorbas-Los Pilares | 1 | 2009 | 2011 |
| Andalusia | Granada | Sierra de Baza-Filabres | 2 | 2009 | 2015 |
| Andalusia | Granada | Lomas Padul-Gororón | 2 | 2006 | 2012 |
| Castile-León | Burgos | Casanova | 4 | 2005 | 2013 |
| Castile-León | Soria | Medinaceli-Adradas | 4 | 2006 | 2013 |
| Castile-León | Soria | Medinaceli-Layna Obetago | 13 | 2006 | 2014 |
| Castile-León | Soria | Medinaceli-Miño Yelo | 5 | 2006 | 2012 |
| Castile-León | Soria | Medinaceli-Radona | 4 | 2006 | 2015 |
| Castile-La Mancha | Albacete | Viveros | 1 | 2005 | 2008 |
| Castile-La Mancha | Guadalajara | Molina-Loma de la Fuente | 1 | 2009 | 2012 |
| Castile-La Mancha | Guadalajara | Molina-WF Loma Gorda | 7 | 2009 | 2013 |
| Castile-La Mancha | Guadalajara | Molina-WFPeñaII | 1 | 2009 | 2011 |
| Castile-La Mancha | Guadalajara | Molina-WFPicazo | 4 | 2009 | 2011 |
| Community of Valencia | Valencia | Ademuz-Pinar2 | 2 | 2004 | 2012 |
| Community of Valencia | Valencia | Ademuz-Pinar3 | 5 | 2004 | 2012 |
| Community of Valencia | Valencia | Ademuz-Tovedas | 1 | 2004 | 2012 |
| Navarre | Navarra | Aeródromo | 1 | 2004 | 2015 |
