# Supplementary material for: European population trends and current conservation status of an endangered steppe-bird species: the Dupont’s lark Chersophilus duponti
Source: PeerJ. 2018 Sep 19;6:e5627. doi: 10.7717/peerj.5627 (PMC6151120; doi:10.7717/peerj.5627)
Supplement: Supplemental Information 4 — Wald-tests and associated p-values (p) are shown. For each period, the annual change rate, the associated 95% Confidence Interval (CI95%) and trend classification attending to TRIM criteria (TRIM Trend; Pannekoek & Van Strien, 2006a) are shown. AN: Andalusia. AR: Aragon. CA: Catalonia. CL: Castile-Leon. CM: Castile-La Mancha. CV: Community of Valencia. NA: Navarre. RM: Region of Murcia. [file peerj-06-5627-s004.docx]

| **Autonomous Community** | **Period** | **Wald-test** | **p** | **Annual change rate (%)** | **CI95%** | **TRIM Trend** |
| --- | --- | --- | --- | --- | --- | --- |
| AN | 2004 – 2006 | 13.53 | < 0.001 | -37.8 | [-53.5; -22.1] | Steep decline |
| AN | 2006 – 2010 | 12.14 | < 0.001 | +13.0 | [-0.3; +26.3] | Uncertain |
| AN | 2010 – 2011 | 4.56 | < 0.05 | -38.1 | [-68.7; -7.5] | Steep decline |
| AN | 2011 – 2012 | 2.07 | < 0.2 | +24.0 | [-44.5; 92.5] | Uncertain |
| AN | 2012 – 2013 | 5.13 | < 0.05 | -75.9 | [-104.8; -47.0] | Steep decline |
| AN | 2013 – 2015 | 4.30 | < 0.05 | +60.4 | [-41.6; +162.5] | Uncertain |
| AR | 2004 – 2015 | 0.59 | > 0.05 | +1.5 | [-2.3; +5.2] | Uncertain |
| CA | 2004 – 2015 | 0.04 | > 0.05 | +3.6 | [-33.5; +40.8] | Uncertain |
| CL | 2004 – 2008 | - | - | 0.0 | - | Stable |
| CL | 2008 – 2009 | 2.00 | < 0.2 | +12.5 | [-5.9; +31.0] | Uncertain |
| CL | 2009 – 2010 | 22.14 | < 0.001 | -45.5 | [-55.9; -35.0] | Steep decline |
| CL | 2010 – 2012 | 17.24 | < 0.001 | -2.4 | [-13.6; +8.7] | Uncertain |
| CL | 2012 – 2013 | 2.03 | < 0.2 | -22.8 | [-42.4; -3.2] | Moderate decline |
| CL | 2013 – 2015 | 2.67 | < 0.2 | +4.5 | [-10.4; +19.4] | Uncertain |
| CM | 2004 – 2005 | 11.01 | < 0.001 | -76.0 | [-96.3; -55.8] | Steep decline |
| CM | 2005 – 2006 | 6.45 | < 0.05 | +122.2 | [-74.3; +298.7] | Uncertain |
| CM | 2006 – 2009 | 2.18 | < 0.2 | +4.6 | [-9.0; +18.2] | Uncertain |
| CM | 2009 – 2010 | 6.02 | < 0.05 | -33.4 | [-52.3; -14.4] | Steep decline |
| CM | 2010 – 2012 | 15.41 | < 0.001 | +40.2 | [+22.6; +57.7] | Strong increase |
| CM | 2012 – 2014 | 12.37 | < 0.001 | -1.7 | [-11.8; +8.4] | Uncertain |
| CM | 2014 – 2015 | 4.05 | < 0.05 | -55.7 | [-73.3; -15.2] | Steep decline |
| CV | 2004 – 2006 | 2.94 | < 0.1 | -25.4 | [-50.3; -0.4] | Moderate decline |
| CV | 2006 – 2007 | 6.79 | < 0.01 | +144.8 | [-1.5; +291.1] | Uncertain |
| CV | 2007 – 2011 | 11.51 | < 0.001 | -16.1 | [-22.2; -9.9] | Steep decline |
| CV | 2011 – 2015 | 7.11 | < 0.01 | +2.0 | [-6.9; +10.8] | Uncertain |
| NA | 2004 – 2015 | 0.11 | > 0.05 | +1.1 | [-7.9; +5.6] | Uncertain |
| RM | 2004 – 2005 | 5.46 | < 0.05 | +125.0 | [-28.0; +278.0] | Uncertain |
| RM | 2005 – 2006 | 12.47 | < 0.001 | -74.1 | [-95.6; -52.5] | Steep decline |
| RM | 2006 – 2008 | 9.90 | < 0.01 | +72.9 | [+4.4; +141.3] | Moderate increase |
| RM | 2008 – 2015 | 7.28 | < 0.01 | -3.3 | [-10.5; +3.8] | Uncertain |
